# Spatially structured multi-wave-mixing induced nonlinear absorption and gain in a semiconductor quantum well

**DOI:** 10.1038/s41598-022-26140-y

**Published:** 2022-12-26

**Authors:** Pradipta Panchadhyayee, Bibhas Kumar Dutta

**Affiliations:** 1 Department of Physics (UG & PG), Prabhat Kumar College (Vidyasagar University), Contai, Purba Medinipur, 721404 India; 2 Department of Physics, Sree Chaitanya College (WB State University), North 24 Parganas, Habra, WB 743 268 India

**Keywords:** Optical physics, Atomic and molecular interactions with photons

## Abstract

We have studied two-dimensional absorption and gain spectrum in an asymmetric semiconductor triple-coupled-quantum-well (TCQW) nanostructure. Four subband transitions are coupled by using four coherent fields in a close-loop configuration to introduce cross-Kerr effect and four-wave-mixing (FWM) induced nonlinearity in achieving nonlinear absorption and gain profiles. Position-dependent absorption and gain are obtained by applying one, or two coherent fields in a variety of standing wave configurations including superposed field configuration in the standing-wave regime. In addition to the control parameters like Rabi frequency and detuning, the specialty of the model is to employ double-controlled spatial phase-coherence guided by the FWM-induced phase and the phases introduced by the standing wave formation. Our results highlight the high-precision electron localization in spatial domain. The evolution of spatially modulated gain without inversion may be a substitute for obtaining gain from a traditional quantum cascade laser. The importance of the present work is to find its application in designing electro-optic modulators in semiconductor nanostructures in near future.

## Introduction

In multi-level atomic systems, laser induced quantum coherence and interference have led to the fundamental quantum-optical effects like coherent population trapping (CPT), electromagnetically induced transparency (EIT), gain without inversion (GWI) and vacuum induced coherence (VIC)^1,2^. Based on these phenomena, it has been possible to explore nonlinear light generation^3–6^, coherent control of absorption and transparency at multi-photon resonance^7–13^ in association with phase dependent characteristics in closed-loop interaction^14,15^, subluminal and superluminal light propagation^16–18^ and all-optical switching of light^19,20^.

On the contrary, laser induced coherence and interference effects have been extensively studied in semiconductor quantum well (SQW)^21–40^. More specifically, electromagnetically induced transparency (EIT)^24,25,33^, gain without inversion (GWI)^21–23^, optical bistability^26,27^, Kerr nonlinearity^28,38^, optical soliton^29^, ultrafast all-optical switching^30^ and other interesting phenomena^31,32,34–37,39,40^, which find applications in optoelectronics and quantum information science. In comparison to the atomic system, using SQW to study the coherence effects has many advantages because of the following reasons: (i) electric dipole moment of intersubband transition in SQW is large due to small effective mass of the electron, (ii) there are flexibilities in designing devices by choosing material and dimensions of the structure, (iii) high nonlinear optical coefficients and (iv) the transition energies, the dipoles and the symmetries can be engineered as per choice^26,27^.

All the effects as mentioned above in atomic systems (SQW) have been studied by considering the coherent interaction of the atom (electron) with the travelling wave field. If one, or more control fields operating in the atomic system (SQW) be considered in standing wave configuration, it would be possible to study the positional confinement of the atom (electron) in one dimension (1D) and two dimensions (2D) as well. In the last two decades, precise position measurement of the atom has attracted a lot of attention because of its potential application in trapping of neutral atoms in laser cooling^41^ and atom nanolithography^42^. Owing to spatially modulated coherence rendered by the standing wave field, a number of works has been proposed on atom localization in 1D^43–53^ and 2D^52–64^, which describe measurement-induced atom localization based on atomic population^45,51,54^, CPT^48^, EIT^61^, interacting dark resonances^49,58^, resonance fluorescence^44,52^, spontaneous emission^46,59^, probe absorption^47,50,53,55–57,60,62,63^ and so on. 2D electron localisation has also been investigated by measuring probe absorption as proposed in ref.^65–70^.

**Figure 1:**
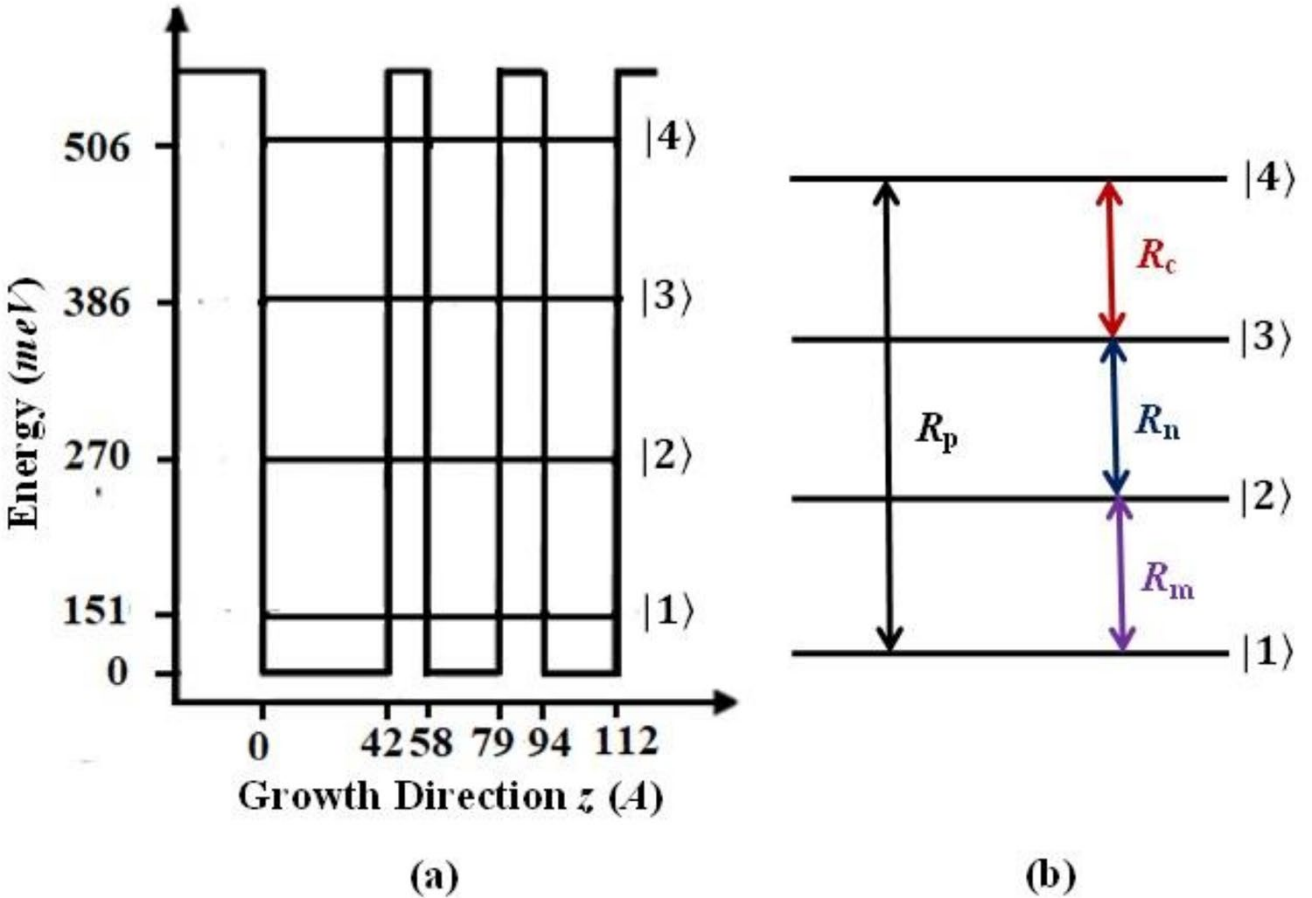
**a** Schematic view of the energy-band diagram of the asymmetric AlInAs/GaInAs TCQW structure (one period) (**b**). Field-coupled energy level diagram of this four-level model (**c**).

In this article, we have chosen an asymmetric semiconductor TCQW nanostructure^68^ leading to four subbands as shown in Fig. 1a. Four subbands are intercoupled by four coherent fields in close-loop-interaction configuration as evident from Fig. 1b. To specify the difference of our model from the theoretical model as described in Ref.^68^, we mention that (i) the way of applying four coherent fields operating in the transitions is different, (ii) the standing wave configurations used in the schemes employed are different, (iii) dual phase controlled mechanism as adopted in this work is specific to the model, and (iv) Semiclassical density-matrix approach has been undertaken in the present model to obtain the expression of probed susceptibility with the nonlinear terms. Individual contributions of cross-Kerr nonlinearity and FWM process are shown in obtaining spatially modulated gain and absorption at various parametric regimes. As a result of FWM-process and standing wave field configuration, double-controlled spatial phase coherence is achieved in our model. We have studied the position dependent probe absorption and gain spectrum by introducing a variety of standing wave fields including superposed field configuration in standing wave regime. Measurement of spatially modulated absorption may lead to high-precision electron localization, which may be found to have applications in controlling electron dynamics in semiconductors. In the present work we have emphasized to obtain high-precision gain as a result of spatially modulated coherence controlled by various field configurations.

Instead of atomic vapour the potentiality of semiconductor nanostructures can be explored to show several strange quantum effects treating the subbands as the electron states of ‘artificial atoms’. All such energy states can be modified by bandgap engineering of the semiconductor nanostructures as well as proper tailoring of the control parameters of systems. This facility promotes the wide use of semiconductor nanostructures in realising exotic quantum features in reality. The novelty of the present work lies in the prominent occurrence of the gain without inversion under spatially modulated coherence, which is an interesting outcome specific to this model over others. Our study stresses on the exploration of new avenues to obtain GWI through the optimum control of the different combinations of standing wave fields and traveling wave fields. Thus, the competition between the position dependent and non-position dependent coherence effects in the close-loop configuration introduces the dominance of the nonlinear coherence effects over linear coherence contribution to the probe susceptibility and consequently generates GWI. Double-controlled spatial phase coherence is achieved in our model due to collective contribution of cross-Kerr nonlinearity and the nonlinearity induced by FWM in different field configurations. In the work we have shown the localization of the high-precision single gain peak in $kx-ky$ domain with 100% detection probability. Control of the spatial position of the occurrence of the single gain peak is shown to be possible by changing the control knobs of spatially modulated coherence. This is worth noting that atom localization can be experimentally realized in laser cooled environment^71^ at the temperature less than micro-Kelvin, while electron or gain localization can be obtained in the semiconductor nanostructure at ambient temperature condition. In quantum cascade lasers, to obtain and maintain population inversion between the lasing states, especially at relatively high temperatures, is a very challenging task because temperature dependent scattering processes reduce the necessary population inversion. In this connection, a general idea for generation of population inversion between the lasing states at low temperature controlled by cryogenic cooling system deals with the application of an external electric field. But, for our system under cryogenic cooling atmosphere, gain is achieved without population inversion by effective control of spatially modulated coherence, which can open up a new way of obtaining gain in semiconductor nanostructure.

## Theoretical model

The TCQW nanostructure is constituted by periodic arrangement of AlInAs (barrier region) and GaInAs (well region) where the layer thicknesses are unequal. Figure 1a shows the schematic energy-band diagram of a single period of this asymmetric structure. The layer thicknesses in the QW regions are 42A (GaInAs well), 16A (AlInAs barrier), 20A (GaInAs well), 16A (AlInAs barrier), and 18A (GaInAs well). The field-coupled energy-level configuration of the four sub-band TCQW structure is shown in Fig. 1b, where the transitions $|1>$-$|2>, |1>$-$|4>, |2>$-$|3>$ and $|3>$-$|4>$ are dipole allowed. In general, fields are considered classically and defined as $E_j(t)=\frac{{\bar{\epsilon }}_j}{2}e^{i\omega _jt}$ + *c*.*c*. ($j=m,n,c,p$), where ${\epsilon _j}/2$ is the field amplitude and $\omega _j$ is the angular frequency. The transitions $|1>$-$|2>$, $|2>$-$|3>$ and $|3>$-$|4>$ are coupled by the three coherent fields with Rabi frequencies denoted as $R_m=\frac{{{{\bar{\mu }}}}_{21}.{{\bar{\epsilon }}}_m}{2\hbar }$, $R_n=\frac{{{{\bar{\mu }}}}_{32}.{{\bar{\epsilon }}}_n}{2\hbar }$ and $R_c=\frac{{{{\bar{\mu }}}}_{43}.{{\bar{\epsilon }}}_c}{2\hbar }$ respectively. A weak coherent field with Rabi frequency $R_p=\frac{{{{\bar{\mu }}}}_{41}.{{\bar{\epsilon }}}_p}{2\hbar }$ is acting in the transition $|1>$-$|4>$. As this field probes the coherence induced by other coupling fields in the system, it is designated as the probe field. Strength of the field with Rabi frequency $R_m$ is also considered to be weak, such that the population transfer occurring from the ground level to the excited level is insignificant due to this field. Here, $\mu _{jk}$ denotes the dipole moment associated with the corresponding transition. In the present model, we note that the fields with Rabi frequencies $R_p$ and $R_m$ will be treated as travelling wave field, while the others with Rabi frequencies $R_n$ and $R_c$ will be spatially modulated for various standing wave configurations, which will be discussed at the end of the section. In this case, $R_n$ and $R_c$ will incorporate the spatial contribution due to the formation of standing wave field.

**Figure 2:**
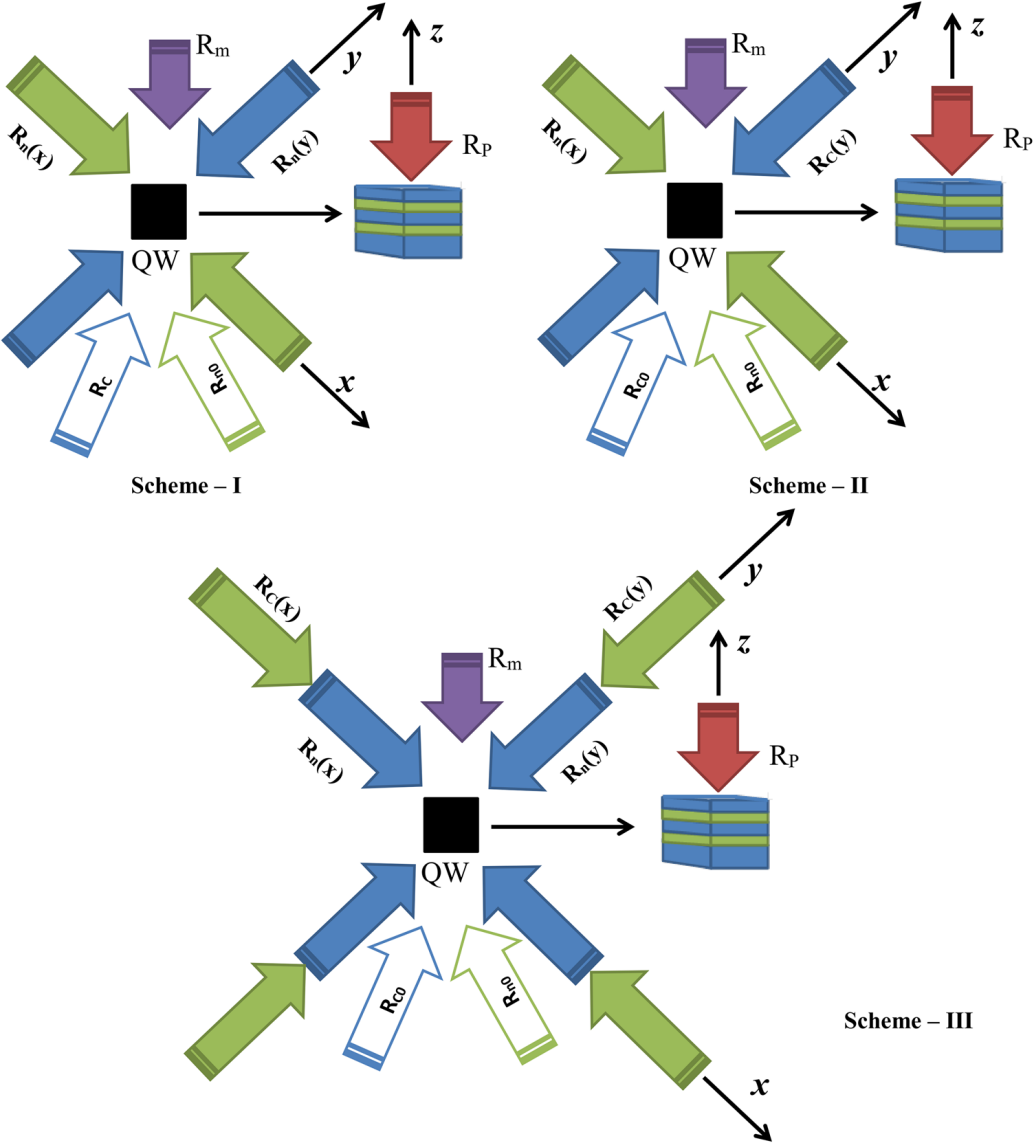
Field configuration related to the asymmetric TCQW structure: three coupling fields ($R_C, R_n$, and $R_m$) are shown in the *x*–*y* plane, where the beam diameters for the three laser fields are greater than the dimension of the SQW system (Well: blue, Barrier: olive) grown along the *z* direction with which the probe field ($R_P$) is applied at an angle 45^∘^. Relevant field components (standing wave fields, and traveling wave fields) are shown for the three schemes.

The coherent part of the atom-field interaction is described by the Hamiltonian under the electric dipole and the rotating wave approximations as

1 $$\begin{aligned} {{\mathcal {H}}}= & {} -{\hbar }[\Delta _m |2> <2| + (\Delta _p - \Delta _c) |3><3| + \Delta _p |4>\nonumber \\{} & {} \quad<4| + (R_m |1> <2| + R_p |1> <4| + R_n |2> <3| + R_c |3> <4| + c.c)] \end{aligned}$$

where $\Delta _m=\Delta _p - \Delta _c - \Delta _n$ with the detuning parameters $\Delta _p=\omega _{p} - \omega _{41}$, $\Delta _c=\omega _{c} - \omega _{43}, \Delta _n=\omega _{n} - \omega _{32}$ and $\Delta _m=\omega _{m} - \omega _{21}$.Here, $\omega _{jk}$ denotes the frequencies of the respective transitions. The system dynamics can be explained by the semiclassical density matrix equation as given by

2 $$\begin{aligned} \frac{\partial {\rho }}{\partial t} = -\frac{i}{\hbar }[{{\mathcal {H}}},\rho ] + \Lambda \rho \end{aligned}$$

where the term $\Lambda \rho$^14^ includes the effect of incoherent decay-mechanism inherent to the present atomic model. The required off-diagonal density matrix equations are presented as follows

3 $$\begin{aligned} {\dot{\rho }}_{41}= & {} -Z_{41} {\rho }_{41} + iR^*_p (\rho _{11} - \rho _{44}) + iR^*_c \rho _{31} -iR^*_m \rho _{42} \end{aligned}$$

4 $$\begin{aligned} {\dot{\rho }}_{31}= & {} -Z_{31} {\rho }_{31} + iR^*_n \rho _{21} + iR_c \rho _{41} - iR^*_m \rho _{32} - iR^*_p \rho _{34} \end{aligned}$$

5 $$\begin{aligned} {\dot{\rho }}_{21}= & {} -Z_{21} {\rho }_{21} + iR^*_m (\rho _{11} - \rho _{22}) + iR_n \rho _{31} - iR^*_p \rho _{24} \end{aligned}$$

where $Z_{41}=\Gamma _{41} - i\Delta _p, Z_{31}=\Gamma _{31} - i(\Delta _p - \Delta _c)$ and $Z_{21}=\Gamma _{21} - i(\Delta _p - \Delta _c - \Delta _n)$. Here, $\Gamma _{21} = (\frac{\gamma _{21}}{2} + \gamma _{21}^d), \Gamma _{31} = (\frac{\gamma _{31}}{2} + \gamma _{31}^d)$ and $\Gamma _{41} = (\frac{\gamma _{41}}{2} + \gamma _{41}^d)$, where $\gamma _{mn}$ ($m=2,3,4$ and $n=1$) denotes the natural decay rate due to longitudinal optical (LO) phonon emission at low temperature^68^ and $\gamma _{mn}^d$ denotes coherence dephasing rate generated from electron-phonon scattering and scattering on interface roughness^34^.

**Figure 3:**
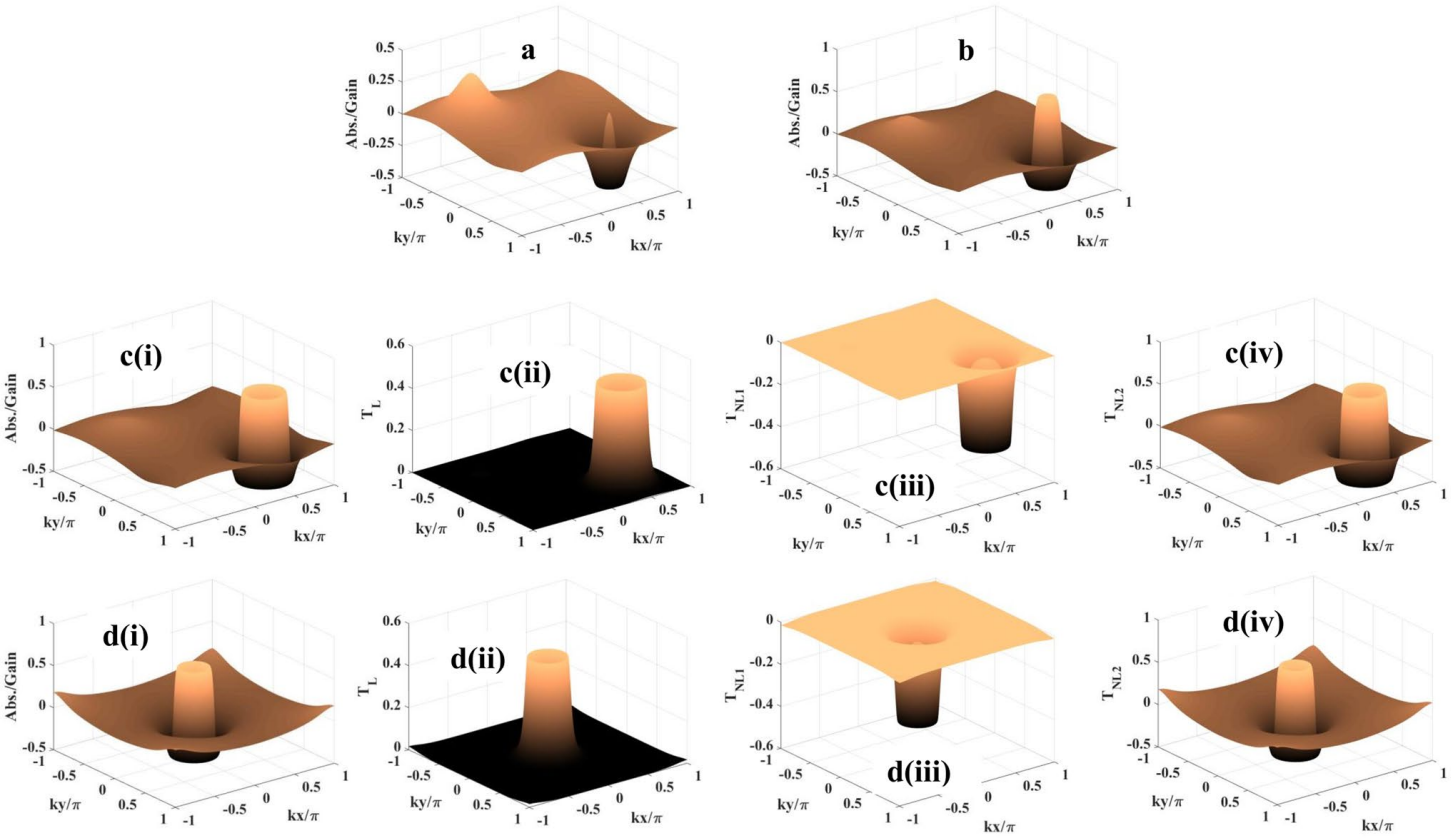
Probe absorption-gain spectra: (**a**) $R_{n0} = 1$ meV, $\phi _1 = \phi _2 = 0$; (**b**) $R_{n0} = 2$ meV, $\phi _1 = \phi _2 = 0$; **c(i)**. $R_{n0} = 4$ meV, $\phi _1 = \phi _2 = 0$; **d(i)** $R_{n0} = 3$ meV, $\phi _1 = \phi _2 =\pi /2$. **c(ii) [d(ii)]**, **c(iii) [d(iii)]**, and **c(iv) [d(iv)]** show the respective contribution of $T_L, T_{NL1}$, and $T_{NL2}$ to the formation of spectrum shown in **Fig. c(i) [d(i)]**. Parameters: $\phi = \pi /2, R_{na} = R_c = 10$ meV, $R_m = 0.05$ meV, $\Delta _p = 30$ meV, $\Delta _c = -10$ meV, and $\Delta _m = 12$ meV.

Under weak-field approximation, we treat the Rabi frequencies $R_p$ and $R_m$ to the first order and the others ($R_n$ and $R_c$) to all orders and the given set of density matrix equations can be solved in steady state to obtain the expression of ${\rho }^{(1)}_{41}$ on the basis of following conditions to be satisfied: $\rho ^{(0)}_{11}\approx 1, \rho ^{(0)}_{42}= \rho ^{(0)}_{32}=\rho ^{(0)}_{34}=0$, and $\rho _{jk}= \rho ^*_{kj}$. By making the substitutions: $\rho ^{(1)}_{41}= {\tilde{\rho }}_{41}e^{-i\phi _p}, R_p=|R_p|e^{i\phi _p}, R_m=|R_m|e^{i\phi _m}, R_n=|R_n|e^{i\phi _n}, R_c=|R_c|e^{i\phi _c}$ with the introduction of collective phase $\phi = \phi _p - \phi _m - \phi _n - \phi _c$, we obtain

6 $$\begin{aligned} \frac{{\tilde{\rho }}_{41}}{|R_p|} = T_L + T_{NL}, \end{aligned}$$

where the linear response of the probe field denoted by the term

$$\begin{aligned} T_L = i\frac{Z_{21}Z_{31}}{Z_{21}Z_{31}Z_{41} + Z_{21}|R_c|^2 + Z_{41}|R_n|^2}, \end{aligned}$$

and nonlinear response denoted by the term $T_{NL}$ given by $T_{NL}=T_{NL1}+T_{NL2}$ with

$$\begin{aligned} T_{NL1}= & {} i\frac{|R_n|^2}{Z_{21}Z_{31}Z_{41} + Z_{21}|R_c|^2 + Z_{41}|R_n|^2}, \\ T_{NL2}= & {} -i\frac{\left[ |R_n||R_m||R_c|/|R_p|\right] e^{i\phi }}{Z_{21}Z_{31}Z_{41} + Z_{21}|R_c|^2 + Z_{41}|R_n|^2}, \end{aligned}$$

where the term $T_{NL1}$ corresponds to the effect of cross-Kerr nonlinearity^72^ and $T_{NL2}$ includes the non-linearity induced by FWM process in the probe response. The second term $T_{NL2}$ holds for the presence of all four fields involved in the model and thereby leading to the signature of four-level loop linkage. As a consequence $T_{NL2}$ represents the appearance of FWM-induced phase coherence through the presence of the phase term $\phi$.

The polarization induced in the probe transition is given by performing the quantum average over the corresponding transition moment^2,6^ as follows

7 $$\begin{aligned} {{\mathcal {P}}}_p = \epsilon _0 \chi _p \epsilon _p = 2N{\mu }_{14}{\tilde{\rho }}_{41} \end{aligned}$$

where $\epsilon _0$ being the free-space permittivity and *N*, the atomic density. The susceptibility $\chi _p$ is expressed as

8 $$\begin{aligned} \chi _p = C \chi \end{aligned}$$

with $C=\frac{N|{\mu }_{14}|^2}{\epsilon _0{\hbar }{\gamma _{41}}}$ as the dimensionless constant^73^ and $\chi = \frac{\gamma _{41}{\tilde{\rho }}_{41}}{|R_p|}$. *C* is equivalent to the weight factor of optical density relating to the susceptibility of probe response. For the sake of simplicity of the calculation, *C* is chosen to be unity. We mention that $Im({\chi })$ and $Re({\chi })$ correspond to probe absorption and dispersion evolved in the system, respectively^1,74^.

*Standing wave field configuration*: In order to study the 2D absorption, or gain, we need to consider one or more Rabi frequencies to be varying spatially. The requirement is fulfilled if we consider the control field in standing wave configuration along *x* and *y* -directions. To illustrate the operation of control field in standing wave regime, we describe the following schemes (Fig. 2) undertaken in the present model.

$Scheme-I$: We redefine the Rabi frequency $R_n$ as

9 $$\begin{aligned} R_n(x,y) = R_{n0} + R_{na} sin(k_nx + {\phi }_1) + R_{na} sin(k_ny + {\phi }_2) \end{aligned}$$

where $R_{n0}$ stands for the travelling wave and the standing wave $R_{na}(x)$ ($R_{na}(y)$) can be directly produced by the counter-propagating wave-vector along the *x*-direction (*y*-direction) and $k_n$ being the propagation vector. The standing wave fields controls the coherent structures of absorption and gain introducing position dependent coherence effect, whereas the traveling wave field acts as a source of non-position dependent coherence effect. Phase-shift introduced by the formation of standing wave component is denoted by $\phi _j$ ($j = 1,2$). Other coupling fields are treated to be travelling wave. In the present scheme, standing wave regime along *x* and *y* -directions is formed by considering single field-coupled transition $|2>$-$|3>$.

$Scheme-II$: In this scheme, we introduce both types (position dependent and non-position dependent) of coherence effects in the transitions corresponding to $R_n$ and $R_c$ of the SQW system. The Rabi frequencies $R_n$ and $R_c$ are redefined as

10 $$\begin{aligned} R_n(x)= & {} R_{n0} + R_{na} sin(k_nx + {\phi }_1), \nonumber \\ R_c(y)= & {} R_{c0} + R_{ca} sin(k_cy + {\phi }_2) \end{aligned}$$

where $R_{c0}$ corresponds to the travelling wave component of $R_c(y)$. $k_c$ denotes the propagation vector. In contrast to the Scheme-I, *x* and *y* directional standing waves are considered for the field-coupled transitions $|2>$-$|3>$ and $|3>$-$|4>$, respectively.

$Scheme-III$: This scheme leads to superposed standing wave configuration for the fields operating in the transitions $|2>$-$|3>$ and $|3>$-$|4>$, which can be envisaged as

11 $$\begin{aligned} R_n(x,y)= & {} R_{n0} + R_{na} sin(k_nx + {\phi }_{n1}) + R_{na} sin(k_ny + {\phi }_{n2}),\nonumber \\ R_c(x,y)= & {} R_{c0} + R_{ca} sin(k_cx + {\phi }_{c1}) + R_{ca} sin(k_cy + {\phi }_{c2}). \end{aligned}$$

In the case of transitions, $|2>$-$|3>$ and $|3>$-$|4>$, the corresponding wavevectors ($k_n$ and $k_c$) related to the standing wave fields are taken as nearly equal .i.e., $k_n = k_c = k$.

**Figure 4:**
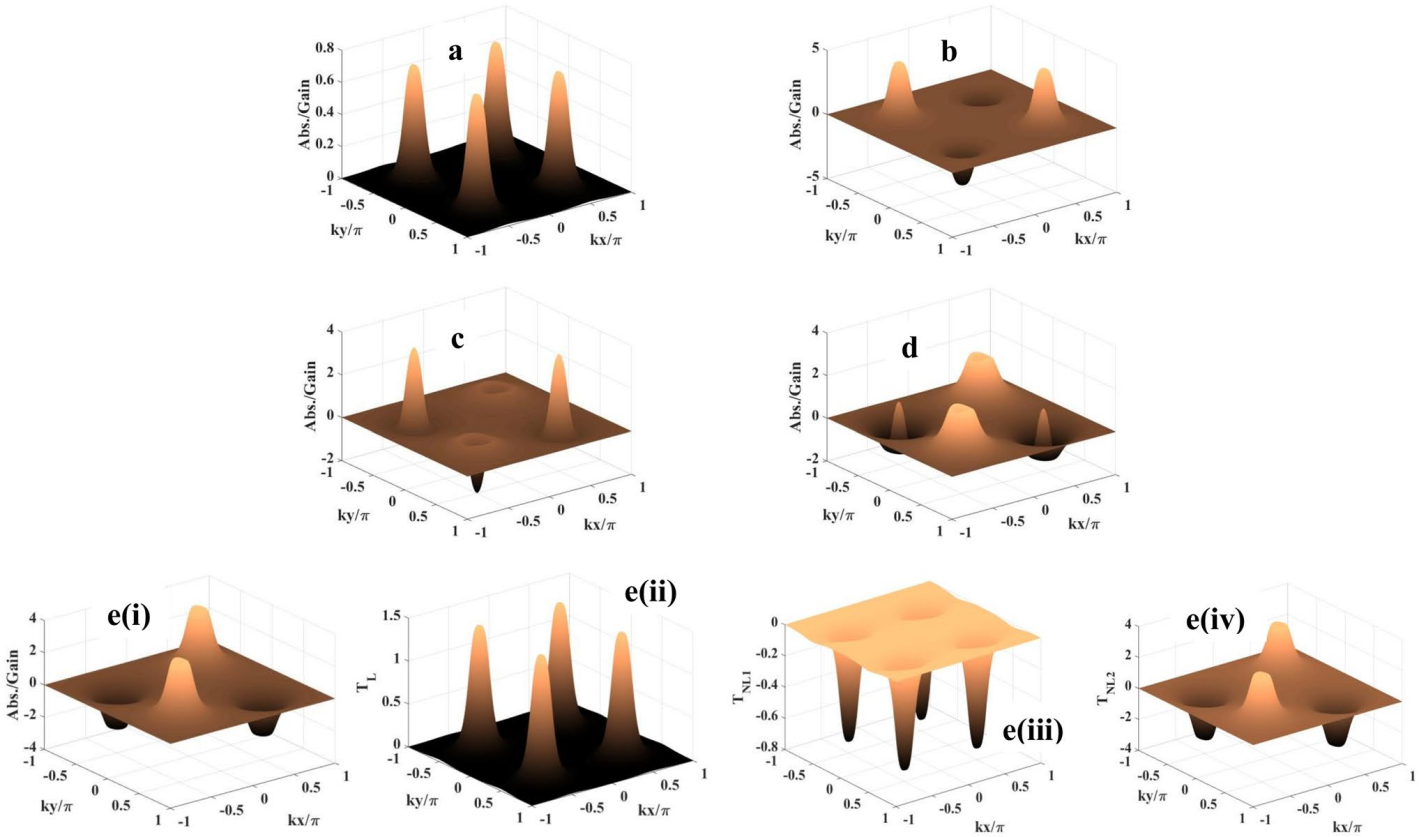
Probe absorption-gain spectra: (**a**) $R_m = 0, \phi = 0$; (**b**) $R_m = 0.05$ meV, $\phi = 0$; (**c**) $R_m = 0.05$ meV, $\phi = \pi /4$; (**d**) $R_m = 0.05$ meV, $\phi = \pi /2$; e(i). $R_m = 0.05$ meV, $\phi = 3\pi /4$. **e(ii)**, **e(iii)** and **e(iv)** show the respective contribution of $T_L, T_{NL1}$, and $T_{NL2}$ to the formation of spectrum shown in **e(i)**. Parameters: $R_{na} = R_{ca} = 10$ meV, $R_{n0} = R_{c0} = 0, \Delta _p = 10$ meV, $\Delta _c = -10$ meV, and $\Delta _m = 10$ meV, $\phi _1 = \phi _2 = 0$.

## Results and discussions

In this section, the 2D probe absorption/gain spectrum ($Im (\chi$) for the AlInAs/GaInAs TCQW structure considered in this model is plotted versus the positions ($kx/\pi , ky/\pi$) and also analyzed to get an insight how the system parameters can be optimized as control knobs. Our main focus of study is to tune suitable parametric conditions for achieving GWI and maximise its detection probability in different spatial positions. The values of $\Gamma _{21}$ , $\Gamma _{31}$ , and $\Gamma _{41}$ are taken as 1.6 meV, 0.65 meV, and 0.5 meV, respectively^68^. The probe Rabi frequency $R_p = 0.01$ meV is set for the whole study. During the whole discussion the units of Rabi frequencies, and detunings are considered in meV units.

At first, in Fig. 3, we investigate and show how the 2D probe absorption/gain depends on the space-dependent control field $R_n(x,y)$ proposed under the Scheme I with the parameters $R_{na} = 10$ meV, $\Delta _p = 30$ meV, $\Delta _c = -10$ meV, and $\Delta _m = 12$ meV. The Rabi frequencies set for the traveling-wave fields applied for the other transitions are $R_c = 10$ meV, $R_m = 0.05$ meV and the magnitude of collective phase is $\pi /2$. For this investigation the traveling-wave part ($R_{na} = 10$ meV) in the field $R_n(x,y)$ is gradually increased accompanied by the phase-shifts ($\phi _1$ and $\phi _2$) introduced by the formation of standing wave components with zero (Fig. 3a–c) and non-zero (Fig. 3d) values. When $R_{n0}$ is applied with the value of 1 meV (Fig. 3a), the circular gain pattern evolves with a central absorption peak at (0.5,0.5) in the first quadrant and a broad absorption zone with the central peak at (-0.5,-0.5) occurs in the third quadrant of the $kx/\pi - ky/\pi$ plane. Although the coherent gain/absorption structure mimics the same as in Fig. 3a when we consider only the FWM-induced nonlinearity ($T_{NL2}$) contribution to compute probe susceptibility, the apperance of the gain peak is attributed to the cumulative nonlinear coherence effect originated due to cross-Kerr nonlinearity ($T_{NL1}$) and the FWM. With the increase of $R_{n0}$ at 2 meV the absoption scenario gets reversed for the two quadrants mentioned in Fig. 3b. In the third quadrant the absorption becomes less pronounced with the decrease in peak height, while the absorption peak assumes greater value and area in the first quadrant. The same type of nonlinear contributions also shapes the pattern shown in Fig. 3b. This feature becomes prominent in Fig. 3c(i) for $R_{n0}$ = 4 meV only with a deviation of the crater-like appearance of gain along with the absorption profile (first quadrant). But the situation drastically changes in Fig. 3d(i) with the incorporation of $\phi _1$ and $\phi _2$ set as $\pi /2$ and $R_{n0}$ = 3 meV. Over the whole wavelength range in *x* and *y* directions gain is achieved with the presence of the central absorption peak with symetrically sagging roof. With an attempt to show the contribution of the linear term ($T_L$) to the formation of gain/absorption profiles shown in Fig. 3c(i) and d(i) we present the corresponding plots in Fig. 3c(ii) and d(ii), respectively. To validate the observation regarding the contribution of the nonlinear terms ($T_{NL1}$ and $T_{NL2}$) to gain/absorption we plot the spectra when $T_{NL1}$ [$T_{NL2}$] only is accounted for the calculation of susceptibility against ($kx/\pi , ky/\pi$) in Fig. 3c(iii) [c(iv)] and 3d(iii) [d(iv)]. It is an important outcome of the present study that the plot of the $T_{NL2}$ term mimics the gain/absorption profiles shown in Fig. 3c(i) and d(i), i.e., the space-dependent 2D manipulation of gain/absorption is made possible as a consequence of the contribution of the nonlinearity induced by FWM. It is to highlight that, in all the cases, the coherent structures arising out of the cross-Kerr nonlinearity ($T_{NL1}$) show only the gain features, whereas the term $T_L$ related to linear coherence effect always exhibits absorption phenomenon. Looking at the term $Z_{41}|R_n|^2$ of the denominators of the $T_{NL}$ expressions the above-mentioned features can be explained on the basis of the change of spatially modulated coherence modified by the coherence induced as a consequence of increasing the travelling wave part of $R_n(x,y)$. The change in $R_{n0}$ sets an interplay between the magnitude of positive and negative contributions of the nonlinear terms in generating 2D gain/absorption patterns. Further, in all the sub-figures of Fig. 3, we observe the sudden change of absorption (gain) closely surrounded by gain (absorption) in 2D domain, which appears as a dispersion-like feature controlled by spatially modulated coherence. There by, it strongly bears a signature of Fano-like quantum interference in spatial domain.

With a view to studying the combined effect of the magnitude of the field $R_m$ ($|1>$-$|2>$ transition) and the collective phase $\phi$ on gain/absorption spectrum for the field configuration proposed in the Scheme II and the corresponding results are plotted in Fig. 4. The parameters: $R_{na} = R_{ca} = 10$ meV, $\Delta _p = \Delta _m = 10$ meV, $\Delta _c = -10$ meV. It is to note that there is no traveling-wave component in the field arrangement of $R_n$ and $R_c$ i.e., $R_{n0} = R_{c0} = 0$. When these two control knobs are in ‘switched-off’ condition, only spatially modulated coherence due to the presence of $R_n(x)$ and $R_c(y)$ comes into play originating one absorption peak at the centre of each quadrant (Fig. 4a). All of them are of same size and height. In this case, the spatial positions of probe absorption maxima are determined by the conditions, $kx + ky = m\pi$ or $kx - ky = n\pi$ ($m \ne n$; *m*, *n* are positive and negative integers including zero). At this condition, only $T_{NL1}$ acts as the nonlinear term which shapes the probe response. When $R_m$ is switched on to a low value of 0.05 meV (Fig. 4b), the absorption peaks are found to be replaced by gain peaks at the centres of the second and the fourth quadrants as a result of combined nonlinear contribution by the $T_{NL1}$ and $T_{NL2}$ terms. This feature remains unaltered but appears with increasing sharpness in absorption peaks and decrease in height of the gain peaks when $\phi$ is set to $\pi /4$ (Fig. 4c). This change in coherent structures of gain and absorption is simply attributed to the introduction of the collective phase ($\phi$). A substantial change in pattern is found for the case of $\phi = \pi /2$ (Fig. 4d). The gain-surrounded absorption peaks are observed in the centres of the first and third quadrants, while the inverted patterns evolve in the other quadrants. The almost inverted pattern with nearly same magnitudes is visible in Fig. 4e(i) ($\phi = 3\pi /4$) as compared to Fig. 4c ($\phi = \pi /4$). The role of the linear term and the nonlinear terms in the evolution of gain/absorption feature as shown in Fig. 4e(i) is also verified and respectively presented in Fig. 4e(ii) and e(iii–iv). The linear contribution ($T_L$) to the susceptibility is shown to be responsible in generating only absorption, as is found in Fig. 3c(ii) and d(ii). The presence of the collective phase ($\phi$) in the nonlinear term ( $T_{NL2}$) is one of the main factors in modulating spatially modulated coherence.

**Figure 5:**
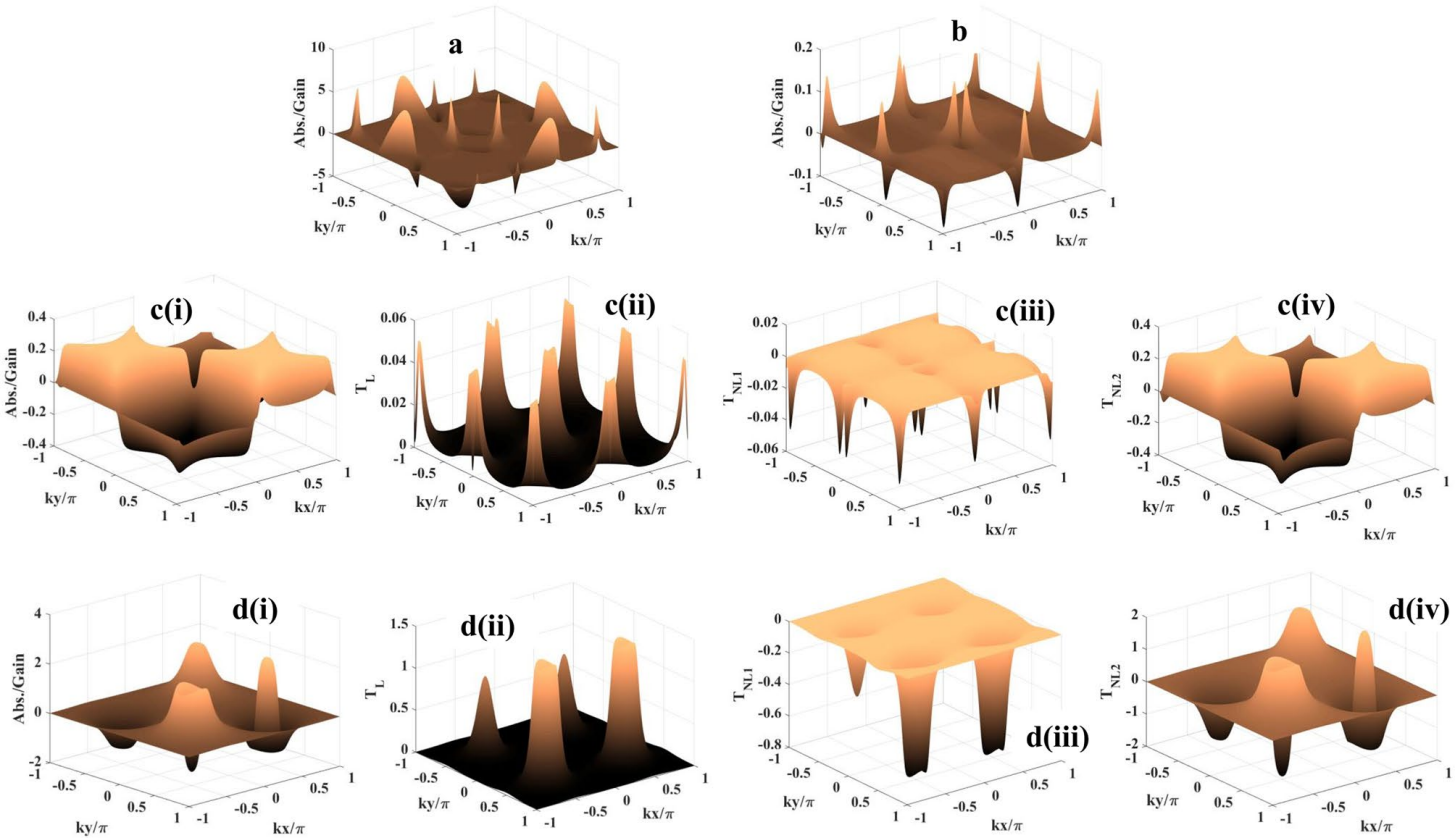
Probe absorption-gain spectra: (**a**) $\phi = 0, \Delta _p = \Delta _m = 5$ meV, $\Delta _c = -5$ meV; (**b**) $\phi = 0, \Delta _p = \Delta _m = \Delta _c = 10$ meV; (**c**) $\phi = \pi /2, \Delta _p = \Delta _m = \Delta _c = 10$ meV; (**d**) $\phi = \pi /2, \Delta _p = \Delta _m = 10$ meV, $\Delta _c = -10$ meV. **c(ii) [d(ii)]**, **c(iii) [d(iii)]**, and **c(iv) [d(iv)]** show the respective contribution of $T_L, T_{NL1}$, and $T_{NL2}$ to the formation of spectrum shown in **c(i) [d(i)]**. Parameters: $R_{na} = R_{ca} = 10$ meV, $R_m = 0.05$ meV, $R_{n0} = 0.5$ meV, $R_{c0} = 0, \phi _1 = \phi _2 = 0$.

The next part of investigation is to study the cumulative effect of the variations in detunings and the collective phases in the presence of the traveling-wave component ($R_{n0}$) with the standing-wave field arrangement $R_n$. Based on such study we plot the gain-absorption profile in Fig. 5 under the field arrangement of the Scheme II. The Rabi frequency parameters are same as those of Fig. 4 except $R_m = 0.05$ meV and $R_{n0}$ = 0.5 meV. In the case of Fig. 5a under the parameter conditions ($\phi = 0, \Delta _p = \Delta _m = 5$ meV, $\Delta _c = -5$ meV) the probe absorption accompanied by gain is more or less uniformly distributed in four quadrants with crescent patterns. When all the detunings are taken as same ($\Delta _p = \Delta _m = \Delta _c$) and set to 10 meV (Fig. 5b), the absorption and gain spectra get sharper but become less in magnitude as compared with Fig. 5a. With the introduction of the non-zero collective phase ($\phi = \pi /2$) as shown in Fig. 5c(i) the gain/absorption profile suffers a drastic change where two similar nearly square-shaped absorption (gain) regimes occur for the first and third quadrants (the second and fourth quadrants). Applying negative detuning to the $|3>$-$|4>$ transition ($\Delta _c = -10$ meV) an interesting pattern of gain and absorption is generated (Fig. 5d(i)). The first and the third quadrants have a central absorption peak surrounded by the gain region and an extended gain peak, respectively while completely inverted features appear in the neighbouring quadrants (4th with respect to 1st and 2nd with respect to 3rd). Figure 5c(iii) [d(iii)] and c(iv) [d(iv)] are presented to strengthen the finding that the $T_{NL2}$ term is mainly responsible for recurrence of the same features as are prominent in Fig. 5c(i) [d(i)]. As seen in the (ii) parts of Fig. 5c,d, the linear part ($T_L$) of the susceptibility does only matter for the occurence of absorption like previous fearures shown in Figs. 3c(ii), d(ii), and 4e(ii). Also, the Fano-like quantum interference in the system is the origin of the dispersion-like nature of the probe absorption spectra in spatial domain. Another point is to note that same features are observed (not shown) if the traveling-wave component, $R_{c0}$ is powered at a value of 0.5 meV instead of $R_{n0}$.

**Figure 6:**
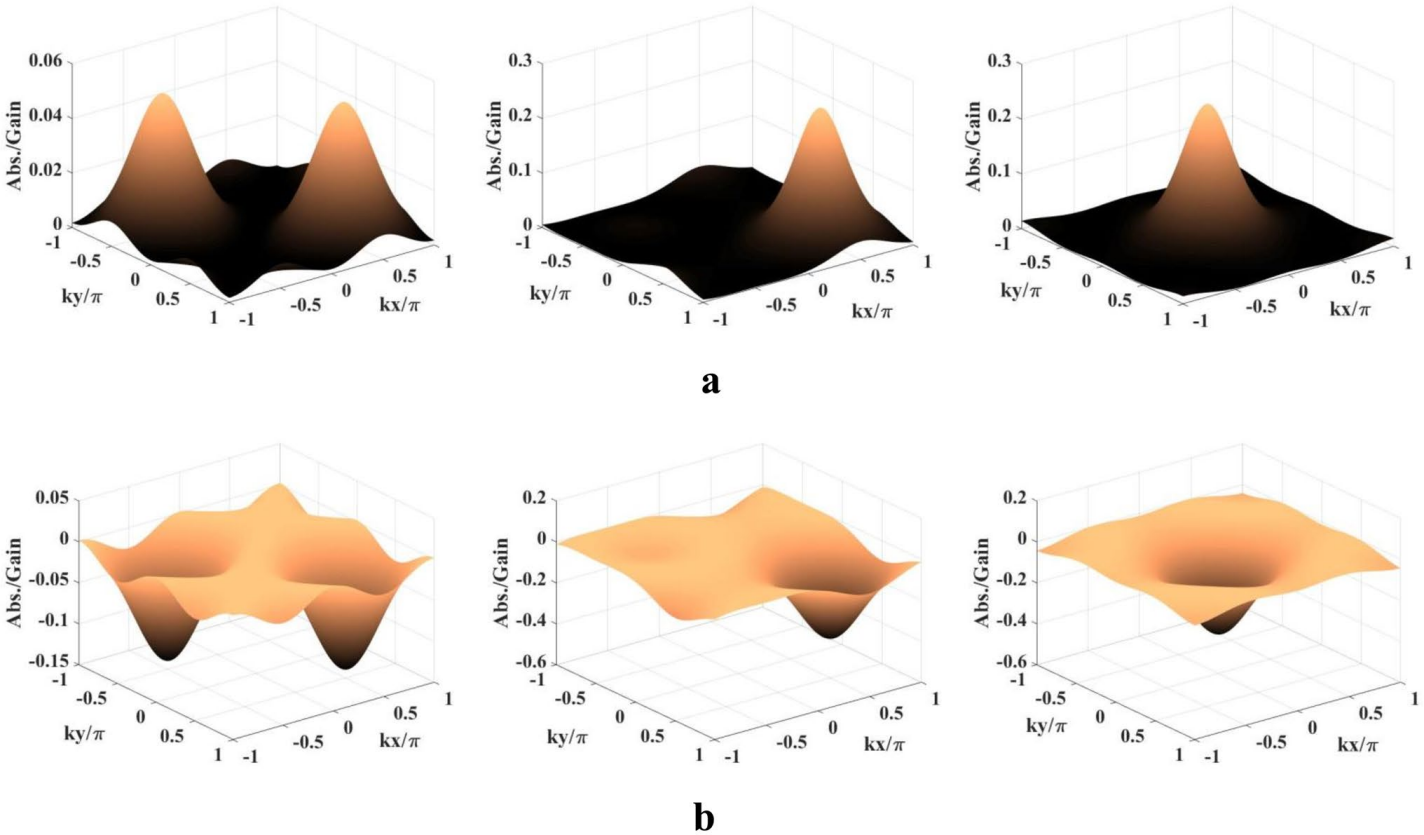
Probe absorption-gain spectra: (**a**) Left - $R_{n0} = R_{c0} = 0, \phi = \phi _{n1} = \phi _{c1} = \phi _{n2} = \phi _{c2} = 0$; Middle—$R_{n0} = R_{c0} = 1.5$ meV, $\phi = \phi _{n1} = \phi _{c1} = \phi _{n2} = \phi _{c2} = 0$; Right—$R_{n0} = R_{c0} = 1.5$ meV, $\phi = 0, \phi _{n1} = \phi _{c1} = \phi _{n2} = \phi _{c2} = \pi /2$. (**b**) Left—$R_{n0} = R_{c0} = 0, \phi = \pi /2, \phi _{n1} = \phi _{c1} = \phi _{n2} = \phi _{c2} = 0$; Middle—$R_{n0} = R_{c0} = 1.5$ meV, $\phi = \pi /2, \phi _{n1} = \phi _{c1} = \phi _{n2} = \phi _{c2} = 0$; Right - $R_{n0} = R_{c0} = 1.5$ meV, $\phi = \phi _{n1} = \phi _{c1} = \phi _{n2} = \phi _{c2} = \pi /2$. Parameters: $R_{na} = R_{ca} = 2$ meV, $R_m = 0.05$ meV, $\Delta _p = 12$ meV, $\Delta _c = -4$ meV, and $\Delta _m = 4$ meV.

**Figure 7:**
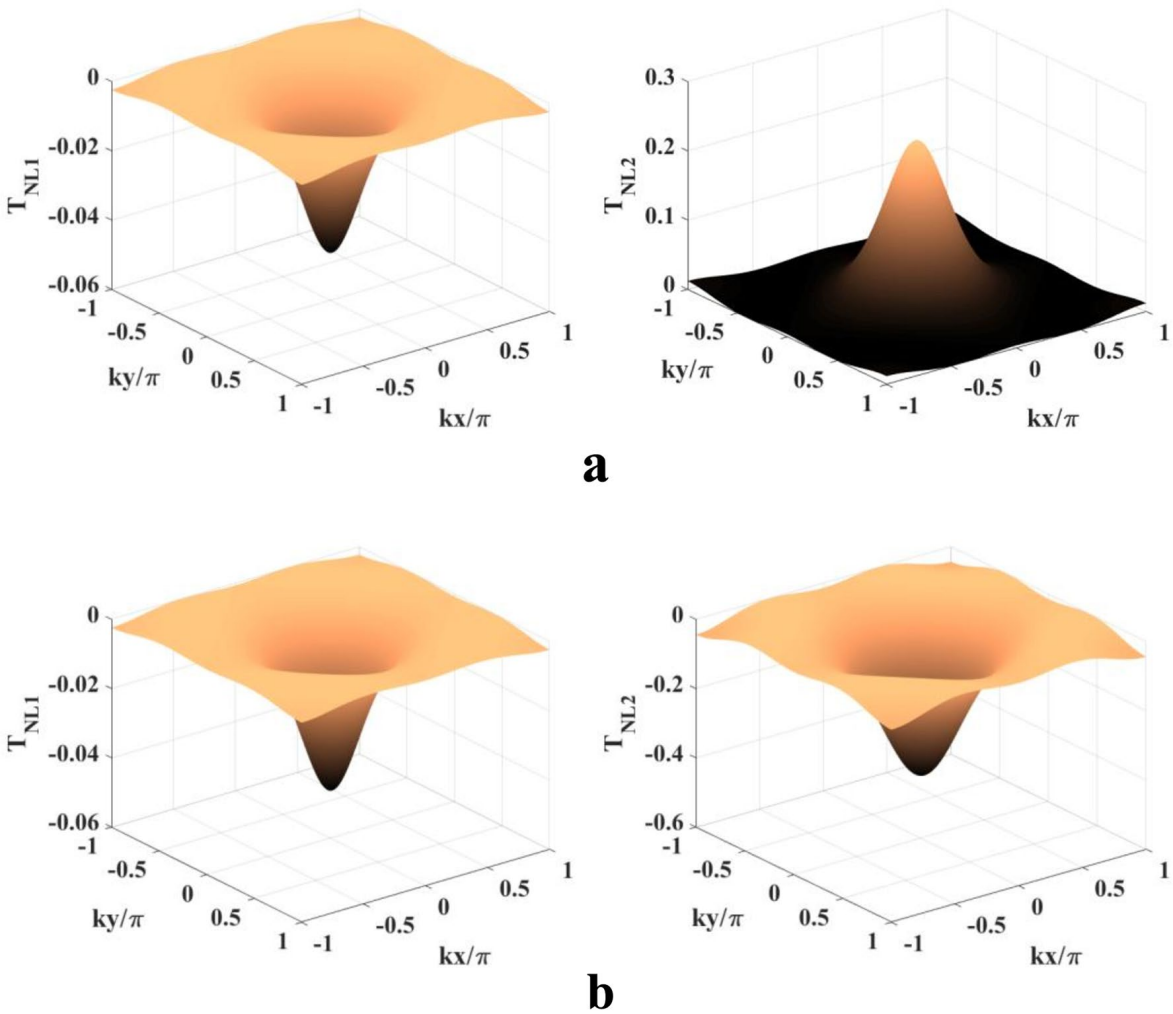
Contribution of the nonlinear terms ($T_{NL1}$ and $T_{NL2}$) to probe absorption-gain spectra of Fig. 6: (**a**) Parameters are same as those for the right panel of Fig. 6a. (**b**) Parameters are same as those for the right panel of Fig. 6b.

In view of the results presented above our final motivation is to find the presence of single absorption/gain (especially GWI) peak as well as to ensure 100% detection probability in the whole domain of *kx*, *ky*. This possibility is found realizable (Fig. 6) following the field configuration as given in the Scheme III. To this aim, we have to tune the traveling-wave components of the two fields ($R_n$ and $R_c$) and also the phase ($\phi$) and phase shifts ($\phi _{n1}, \phi _{n2}, \phi _{c1}, \phi _{c2}$). The parameters are: $R_{na} = R_{ca} = 2$ meV, $R_m = 0.5$ meV, $\Delta _p = 12$ meV, $\Delta _c = -4$ meV, and $\Delta _m = 40$ meV. The evolution of double peaks to the single peak (left and middile panels of Fig. 6a) is observed only by switching on the the traveling-wave parts ($R_{n0} = R_{c0} = 1.5$ meV). This is the natural consequence of the presence of traveling-wave parts which strongly modulates the contribution by the spatially induced coherence. As is shown in the right panel of Fig. 6a the position of the single absorption peak can be modulated by the phase-shift terms and it can be placed at the central position of one-wavelength 2D domain by setting $\phi _{n1} = \phi _{n2} = \phi _{c1} = \phi _{c2} = \pi /2$. As compared to the left panel of Fig. 6a the complete opposite pattern (gain peaks in lieu of absorption peaks) is attainable only assuming the value of collective phase as $\pi /2$. Similar type of manipulation of the gain peak is also exhibited in the middle ($\phi = \pi /2, \phi _{n1} = \phi _{n2} = \phi _{c1} = \phi _{c2} = 0, R_{n0} = R_{c0} = 1.5$ meV) and right ($\phi =\phi _{n1} = \phi _{n2} = \phi _{c1} = \phi _{c2} = \pi /2, R_{n0} = R_{c0} = 1.5$ meV) panels of Fig. 6b. Figure 7 is presented to show again the contributions of the nonlinear terms, $T_{NL1}$ and $T_{NL2}$, to the evolution of the single gain/absorption peak, which is the one of the most intriguing features of our study. It is prominent from Fig. 7 that the positive magitude of $T_{NL2}$ contribution supercedes the negative magnitude of $T_{NL1}$ contribution in evolving the single absorption peak (right panel of Fig. 6a) while both of their contributions with negative magnitude are added leading to the appearance of the single gain peak (right panel of Fig. 6b). We have also examined that the same explanation is applicable to all the panels of Fig. 6a,b.

Finally, for the Figs. 4, 5, 6 and 7, it is examined and validated that all the 2D patterns are mainly the outcomes of the presence of the FWM-induced nonlinear coherence effect related to the $T_{NL2}$ term in the expression of susceptibility and the $T_{NL1}$ term participates partly in the process of generating gain. The linear term $T_L$ contributes to the appearance of absorption only. We highlight that selection of the spatial position of the occurrence of the single gain peak is made plausible by changing the control knobs of spatially modulated coherence.

## Conclusion

We have studied spatially modulated nonlinear effect on the 2D probe absorption-gain in an asymmetric AlInAs/GaInAs TCQW semiconductor nanostructure which may be envisioned as a four-level close-loop interaction system. Four coherent fields are applied and controlled in such a manner that the space-dependent manipulation of absorption and gain is made plausible as a result of combined effect of four-wave-mixing-induced coherence and cross-Kerr nonlinearity. We note that various standing-wave field arrangements with superposed field configuration in standing-wave regime are tuned to originate the several interesting probe absorption-gain patterns. It has been described how single absorption/gain peak over the whole wavelength range in 2D domain is achieved by treating Rabi frequencies and phase factors as control knobs. We have also shown the localization of the single gain peak in $kx-ky$ domain with 100% detection probability. The scope of modulating the spatial position of the occurrence of such high-precision gain may give the experimentalists an impetus to explore the possibility in obtaining GWI for effective lasing in a semiconductor nanostructure at ambient temperature. We emphasize that the FWM-induced nonlinear coherence effect plays the pivotal role in modulating 2D gain/absorption features. It can be highlighted that the way of obtaining gain may be an useful and alternative technique for generation of gain (gain without inversion) which is different from traditional gain mechanism in the case of quantum cascade laser. In the present study, the ambient temperature is to be controlled by cryogenic cooling system to reduce carrier density and thereby minimise the probability of several many-body effects which appear as a hindrance to achieve the goal. Proper tuning of nonlinear modulation effect in such semiconductor nanostructure may also lead to shaping an interesting proposal for a new but efficient electro-optic modulator in coming days.

## Data Availability

The datasets used and/or analysed during the current study available from the corresponding author on reasonable request.
