# Author Correction: Spatially structured multi-wave-mixing induced nonlinear absorption and gain in a semiconductor quantum well

**DOI:** 10.1038/s41598-024-55451-5

**Published:** 2024-02-28

**Authors:** Pradipta Panchadhyayee, Bibhas Kumar Dutta

**Affiliations:** 1 Department of Physics (UG & PG), Prabhat Kumar College (Vidyasagar University), Contai, Purba Medinipur, 721404 India; 2 Department of Physics, Sree Chaitanya College (WB State University), North 24 Parganas, Habra, WB 743 268 India

Correction to: *Scientific Reports* 10.1038/s41598-022-26140-y, published online 26 December 2022

The original version of this Article contained an error in clarification of the constant, *C*. As a result of this error, in the Theoretical model section,

“*C* implies the optical depth of the probe field.”

now reads:

“*C* is equivalent to the weight factor of optical density relating to the susceptibility of probe response.”

The original Article has been corrected.

